# A Lung Organotypic Coculture Reveals a Role for TFEB-Lysosomal Axis in the Survival of Disseminated Dormant Cancer Cells

**DOI:** 10.3390/cancers13051007

**Published:** 2021-02-28

**Authors:** Manuela Zangrossi, Probir Chakravarty, Patrizia Romani, Sirio Dupont, Steven Hooper, Erik Sahai, Marco Montagner

**Affiliations:** 1 Department of Molecular Medicine, University of Padua, Viale G. Colombo, 3, 35126 Padua, Italy; manuela.zangrossi@unipd.it (M.Z.); patrizia.romani@unipd.it (P.R.); sirio.dupont@unipd.it (S.D.); 2 Bioinformatics Platform, Francis Crick Institute, 1 Midland Road, London NW1 1AT, UK; Probir.Chakravarty@crick.ac.uk; 3 Tumor Cell Biology Lab., Francis Crick Institute, 1 Midland Road, London NW1 1AT, UK; steven.hooper@crick.ac.uk

**Keywords:** dormancy, tumor microenvironment, lysosomes, TFEB, in vitro models, organotypic systems

## Abstract

**Simple Summary:**

One of the worst aspects of tumors is the relapse of metastatic lesions several years after the removal of the primary tumor and after the patient has been considered disease-free. This particular aspect is called “metastatic dormancy”. Unfortunately, this behavior is particularly hard to study because disseminated cancer cells are technically not detectable by current imaging techniques. We developed a culture of breast cancer cells and lung epithelial cells that recapitulates in vitro several aspects of what is observed in the real lung. With this tool we identified a specific feature: a lysosomal process that is activated in breast cancer cells and which might be used in the future to target those cells before they wake up.

**Abstract:**

(1) Background: metastatic relapse following a prolonged period of disease-free survival is a common cause of mortality for many cancer patients. Disseminated dormant cancer cells (DDCCs) lie below the radar before waking up years, or even decades, after the removal of the primary tumor. This implies that they are able to survive in a latent state in a foreign environment for an extended period of time supported by intrinsic and extrinsic factors still to be elucidated. (2) Methods: we employed a coculture of DDCCs with lung epithelial cells together with RNA sequencing analysis to understand the overlap in gene transcription between in vivo and cocultured DDCCs. (3) Results: we found a significant overlap between the processes activated in DDCCs from lungs and in the coculture, as well as in alveolar type I cells in vivo and in coculture. We identified the transcription factor EB (TFEB)-lysosomal axis as a relevant process activated in DDCCs upon dissemination to the lung and confirmed the results in our lung coculture. Interestingly, breast cancer patients with a higher expression of TFEB targets show increased likelihood of developing relapses. (4) Conclusions: we propose that lysosomal accumulation following TFEB activation is an important feature of breast cancer DDCCs that might be exploited for future therapeutic interventions.

## 1. Introduction

Metastatic dormancy is a stage during cancer progression in which disseminated cancer cells undergo quiescence, or a proliferation balanced with apoptosis, for prolonged time and possibly undergo proliferative switch leading to metastatic disease. This is not a feature common to all types of cancer [1,2]. For example, breast cancer-positive for estrogen receptor shows increased metastatic likelihood up to 20 years after the removal of the primary tumor, while estrogen receptor-negative subtypes either relapse within the first 5 years or don’t relapse at all [3,4]. Even though this is an old concept, only in the last decade researchers have started to unravel the mechanisms behind metastatic dormancy, mainly thanks to the development of innovative in vitro models [5,6]. As a matter of fact, detection, isolation and characterization of scattered metastatic cancer cells in secondary organs is technically unfeasible. To circumvent this problem, in the last two decades several groups developed techniques to detect disseminating cells (circulating tumor cells, CTC) or tumor biomarkers (such as cell-free tumor DNA) from blood-based biopsies [7,8]. Importantly, several reports showed that the presence of CTCs is a predictor of late recurrence [7]. While CTCs hold the potential to facilitate our understanding of the molecular mechanisms of dissemination and survival, their characterization has been limited by the low concentration of detectable CTCs in patients with cancer in the early stages and high intra-patient heterogeneity. Thus, the design of new organotypic systems to study the processes involved in metastatic dormancy that include different components of the metastatic niche in vitro is of paramount importance. The metastatic niche involves biochemical (oxygen levels, metabolites), biophysical (forces, shear stress, tissue architecture and stiffness) and stromal components (tissue specific cell populations and ECM). Several groups developed in vitro models to study metastatic dormancy by including one or more of the abovementioned components and validated their models according to different criteria [5]. Here we performed a thorough characterization of the transcriptional program of DDCCs and lung epithelial cells in our in vitro system and compared the former with RNAseq data derived from in vivo isolated DDCCs.

Our original lung organotypic system included cellular models of lung parenchymal cells, such as alveolar type I (AT1)-like cells, alveolar type II (AT2)-like cells and immortalized lung fibroblasts on an air-permeable surface and a mitogen low, nutrient low medium (MLNL). Upon culture with this lung coculture, human and mouse DDCCs underwent quiescence and developed several traits in common with DDCCs isolated from mouse lungs, such as cellular protrusions and fibronectin fibrillogenesis [9]. Importantly, we showed that both growth-suppressive and pro-survival signals were released from lung cells. 

Here we show that a simple coculture of DDCCs with AT1-like cells is sufficient to induce a gene expression with a high similarity to that activated by DDCCs upon dissemination to the lungs. Moreover, AT1-like cells exhibit activation of proliferative pathways as previously observed in vivo. These analyses revealed the activation of transcription factor EB (TFEB) signaling and lysosomal accumulation in coculture and in vivo, suggesting a potential new vulnerability of DDCCs. 

## 2. Materials and Methods

### 2.1. Cell Lines

D2.0R and MCF7-GFP cells were a gift from D. Barkan (University of Haifa, Israel), and the T47D-DBM-GFP cells were a gift from R. Gomis (Institute for Research in Biomedicine, Barcelona, Spain). Alveolar type 1-like cells (TT1 cells, a gift from J. Downward, The Francis Crick Institute, London, UK) were originally provided by T. Tetley (Imperial College, London, UK). The generation of D2.0R-EGFP is described in [9]. All cells were cultured in DMEM with 10% FBS (Thermo Fisher Scientific, Waltham, MA, USA, 41965-039) and routinely screened for mycoplasma at the Cell Services facility at The Francis Crick Institute or with a Universal Mycoplasma Detection kit (ATCC, Manassas, VA, USA, 30-1012 K).

### 2.2. Lung Organotypic System

The Coculture was prepared for survival analysis and imaging as follows: 1.36 × 10^5^ TT1 cells/well were plated in MLNL medium (low-glucose DMEM (Thermo Fisher Scientific, Waltham, MA, USA, 21885025), 1% FCS) on a Lumox 24-multiwell plate (Sarstedt, Nümbrecht, Germany, 94.699.00.14). After 24 h, breast cancer cells (100 cells/well for survival assays, 500 cells/well for imaging) were added to the TT1 cell layer. For survival assays, GFP-positive cells were manually counted under an inverted fluorescent microscope after 4 days in coculture. For RNA sequencing, 1.36 × 10^6^ AT1-like cells/sample were plated in 60 mm dish in a MLNL medium; the next day, 1.8 × 10^5^ D2.0R-EGFP cells/sample were added (three biological replicates/sample). On day 3 of coculture, total RNA was extracted using RNeasy Plus Micro Kit (Qiagen, Hilden, Germany) after separation of EGFP-positive and EGFP-negative (AT1-like cells) by Fluorescence-Activated Cell Sorter (FACS).

### 2.3. Lysosomes and Autophagic Flux Visualization

Cocultures were prepared onto coverslips as described above. After 48 h, LysoTracker Red DND-99 (Thermo Fisher Scientific, Waltham, MA, USA, L7528) was added to the culture medium to a final concentration of 50 nM and incubated at 37 °C for 30 min. Coverslips were mounted with ProLong Diamond Antifade Mountant with DAPI (Invitrogen, Carlsbad, CA, USA, P36962) after fixation in 4% PFA for 12 min at room temperature.

Images were analyzed with Fiji software (https://imagej.net/Fiji (accessed on 27 February 2021)) and Lysotracker+ area was determined with the “Analyze particles” tool after applying the same threshold to binarized images. We first generated two selections, one of the whole cell surface (according to GFP) and the other of the nuclear surface (according to DAPI). The percentage of Lysotracker+ cytoplasmic area was calculated according to the formula Lysotracker+ area/(total cell area-nuclear area)*100. For quantification, at least 20 fields were acquired for each condition using the same acquisition settings.

Parental D2.0R cells were transfected with a pCNA3.1-LC3-mCherry-GFP plasmid by using the Lipofectamine 3000 Transfection Reagent (Invitrogen, Carlsbad, CA, USA, L3000001) following manufacturer’s instructions. The coding sequence for the fusion protein [10] was subcloned via Gateway cloning into pCDNA3.1+. One day after transfection, D2.0R cells were plated on a 13 mm coverslip either on AT1-like layer (coculture, 1.5 × 10^3^ D2.0R cells/well onto 1.36 × 10^5^ TT1 cells/well plated in MLNL medium the day before) or on plastic (monoculture, 1.5 × 10^4^ D2.0R cells/well). After 48 h, cells were fixed on ice cold FA 4% for 15 min and then washed with PBS. Treatment with 100 μM Chloroquine (Sigma-Aldrich, St. Louis, MO, USA, C6628) for 3 h prior to fixing was used as control. The coverslips were mounted with ProLong Diamond Antifade Mountant with DAPI (Invitrogen, Carlsbad, CA, USA, P36962). Images were acquired with Leica Stellaris confocal microscope employing the LasX software (63x objective). Quantification was performed by using ImageJ. Single stacks images were converted into 8-bit format and the threshold was adjusted to avoid background signal. The mCherry and GFP puncta were counted by using the “Analyze particles” tool with a filter of 0.4 pixel. Pearson’s correlation coefficient was calculated by using the colocalization function. The autophagic flux is expressed as a mean of the ratio between the number of mCherry puncta divided by the area of the cytoplasm (excluding the nucleus) of a single cell.

### 2.4. Bioinformatic Analysis

RNA sequencing. Prior to analysis, the quality of the RNA samples was assessed using the NanoDrop 8000 spectrophotometer v.2.0 (Thermo Fisher Scientific, Waltham, MA, USA, for quantity) and Agilent 2100 Bioanalyser (Agilent Technologies, Santa Clara, CA, USA, for integrity). Biological replicate libraries were prepared using the polyA KAPA mRNA HyperPrep Kit and sequenced on Illumina HiSeq 4000 platform, generating ~24 million 100 bp single-end reads *per* sample. Read-quality trimming and adaptor removal were carried out using Trimmomatic (version 0.36). The RSEM package (version 1.3.30) [11], in conjunction with the STAR alignment algorithm (version 2.5.2a) [12], was used for the mapping and subsequent gene-level counting of the sequenced reads with respect to the Ensembl mouse GRCm.38.89 version transcriptome. The normalization of raw count data and differential expression analysis was performed with the DESeq2 package (version 1.18.1) [13] within the R programming environment (version 3.4.3) [14]. Differentially expressed genes were defined as those showing statistically significant differences (False Discovery Rate (FDR) < 0.05). Differential gene lists ranked by the Wald statistic were used to look for pathways and selected gene sets using the Broad’s Gene Set Enrichment Analysis (GSEA) software (version 2.1.0) with gene sets from MSigDB (version 6) [15] and additional published and custom datasets (Table S1). Spearman’s rank correlation was used to compare the normalized enrichment scores by comparisons with different experiments to determine which pathways were similarly enriched. Scatterplots (generated using the R base graphics package) shows the correlation between the Wald’s statistic (gene level differences from DESeq2) or the normalized enrichment score (NES)(pathway level differences from GSEA) when comparing D2.0R lung-disseminated_vs_monoculture and coculture_vs_monoculture comparisons.

Survival analysis. Kaplan-Meier were generated with KM Plotter online tool (https://kmplot.com/analysis/ (accessed on 27 February 2021)) which calculates log-rank *p* value. Options used: “Use mean expression of selected gene”, “Autoselect best cutoff”, “User selected probe set” and “Derive ER status from gene expression data”.

### 2.5. Growth Assays

Resazurin staining. AT1-like cells were seeded in 96-well plates (2.176 × 10^4^/well in quadruplicate) in parallel with standards in the linear range of detection. 24 h after seeding, 4 nM Bafilomycin A1 (Sigma-Aldrich, St. Louis, MO, USA, B1793) was added with fresh medium. After 4 days of treatments, cells were washed with PBS and incubated with 100 µM Resazurin (Sigma-Aldrich, St. Louis, MO, USA, R7017) in culture medium at 37 °C for 2 h. Absorbance at 544/590 nm was measured on live cells by checking that the signal was in the temporal linear range. An absolute cell number was then calculated based on the standard curve, after background subtraction (medium without cells).

Cell number quantification with Operetta system. Cells were seeded in 96-well plates 5 × 10^4^ cells/well in quadruplicate). Then, 24 h after seeding, culture medium was renewed by adding 1, 2 or 4 nM Bafilomycin A1. After 4 days of treatments, cells were fixed for 10 min in PFA 4% at room temperature, washed in sterile PBS and incubated for 15 min with Hoechst (Life technologies, Carlsbad, CA, USA, H1399, 1 ug/mL). The cells were automatically counted using the Operetta high-content imaging system based on nuclear counterstaining. Live-cell analysis was performed using Harmony high content imaging and analysis software.

### 2.6. Reporter Assay

At day 1, D2.0R cells were transfected with TFEB transcriptional reporter plasmid (RAGD promoter cloned upstream of luciferase gene, a gift from Prof. Graziano Martello, University of Padua, Italy) [16], together with a plasmid with constitutive expression of Renilla luciferase to normalize for transfection efficiency [17] with Lipofectamine 3000 Transfection Reagent (Invitrogen, Carlsbad, CA, USA, L3000001). After 6 h, 1.8 × 10^4^ transfected cells were plated both on a TT1 layer (coculture, 1.36 × 10^5^ cells/well) and on plastic (monoculture) in a 24-well format. Subsequently, 48 h after replating, cells were harvested in Luc lysis buffer (25 mM Tris pH 7.8, 2.5 mM EDTA, 10% glycerol, 1% NP-40) and the samples on plastic were diluted 1:5 in Luc lysis buffer to balance the Luciferase/Renilla content compared to the coculture. Luciferase and Renilla activity were determined in a Tecan plate luminometer with freshly reconstituted assay reagents (0.5 mM D-Luciferin (Sigma-Aldrich, St. Louis, MO, USA, L9504), 20 mM tricine, 1 mM (MgCO_3_)_4_Mg(OH)_2_, 2.7 mM MgSO_4_, 0.1 mM EDTA, 33 mM DTT, 0.27 mM CoA, 0.53 mM ATP for Luciferase reaction, and 4 µg/mL coelenterazine (Invitrogen, Carlsbad, CA, USA, C2944) in TBS 1X for Renilla reaction). Each sample was transfected in at least three biological duplicates in each experiment.

### 2.7. Reverse Transcriptase Real Time PCR (RT-qPCR)

At day 1, AT1-like cells (1.36 × 10^6^ cells/sample) were plated in MLNL medium in 60 mm dishes. At day 2, D2.0R-EGFP cells (1.8 × 10^5^ cells/sample) were plated on top of an epithelial layer or on plastic and cultured for three days before being harvested. Total RNA was extracted using RNeasy Plus Micro Kit (Qiagen Hilden, Germany) from the whole coculture, and retrotranscribed; the mouse genes were amplified by using mouse-specific qPCR primers. In particular, the total RNA was retrotranscribed with dT-primed M-MLV Reverse Transcriptase (Thermo Fisher Scientific, Waltham, MA, USA, 28025013). qPCR analysis was carried out in a QuantStudio 6 Flex Real-Time PCR System (Thermo Fisher Scientific, Waltham, MA, USA) with Fast SYBR Green Master Mix (Applied Biosystems, Foster City, CA, USA, 4385612). D2.0R cells were normalized to GFP expression levels (not expressed in AT1-like cells). The list of primers used in qPCR is provided in Table S3.

### 2.8. Statistical Methodology

The normal distribution of data was tested with Shapiro–Wilk test for experiments with a sample size greater than 10. For sample sizes lower than 10, it is not easy to assess the underlying data distribution, so non-parametric tests were preferred. For samples sizes lower than five, we preferred parametric tests owing to the minimum possible *p* value becoming large in the non-parametric case. For normally distributed samples, we performed Student’s two-tailed *t*-test for single comparisons (paired or unpaired) and ANOVA test (one-way or two-ways) for multiple comparisons. For non-normal data, we performed the two-tailed Mann–Whitney test for single comparisons and the Kruskal–Wallis test for multiple comparisons. Statistical analyses were performed with GraphPad Prism Software. For survival plots (Kaplan–Meier analysis), data were analyzed with a KM Plotter (https://kmplot.com/analysis/ (accessed on 27 February 2021)) online tool, which calculates the log-rank *p* value (Mantel–Cox method). The Gene Set Enrichment Analysis (GSEA) was generated from the GSEA online tool (http://software.broadinstitute.org/gsea/index.jsp (accessed on 27 February 2021)), which also calculated the two primary statistics of the analysis: Normalized Enrichment Score (NES) and False Discovery Rate (FDR). NES is calculated by normalizing the enrichment score to the gene-set size; the FDR represents an estimated likelihood that a gene set with a given NES represents a false positive.

## 3. Results and Discussion

**Figure 1:**
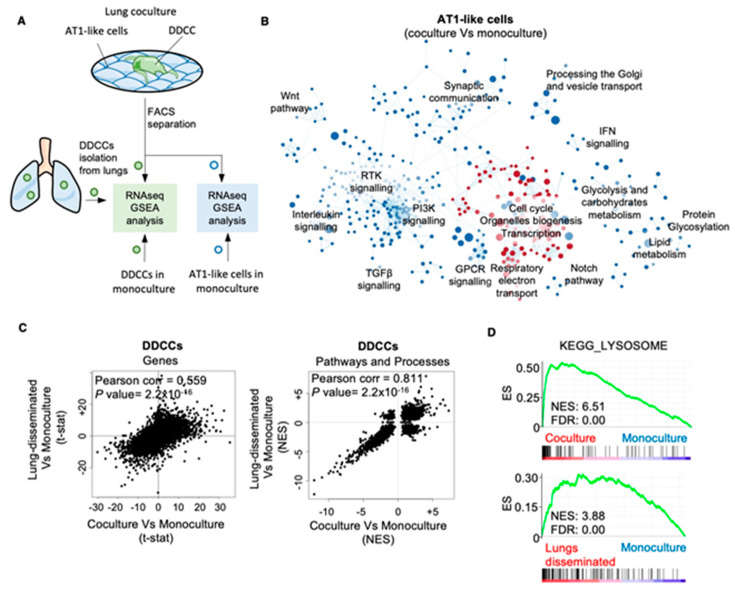
Comparison of the transcriptional programs activated in disseminated dormant cancer cells (DDCCs) from coculture, monoculture and lungs. (**A**) Outline of the transcriptomic analysis. (**B**) Enrichment map of lung AT1-like cells upon coculture with indolent breast cancer cells. The map shows gene-set enrichment results of AT1-like cells cocultured with D2.0R-EGFP cells compared with AT1-like cells in monoculture. Node size, genes in pathway; node color, enrichment score (red indicates enrichment in cocultured AT1-like cells, blue indicates enrichment monocultures of AT1-like cells); edge width, overlap size between connected nodes. (**C**) Scatterplots show the correlation between, left, the Wald’s statistic (gene level differences from DESeq2) and, right, the normalized enrichment score (pathway level differences from GSEA) of D2.0R-EGFP cells disseminated in vivo (lungs), in coculture or monoculture as indicated. (**D**) Profile of the running ES score for KEGG_LYSOSOME gene set after GSEA of D2.0R-EGFP cells disseminated in vivo, in coculture or monoculture as indicated.

Our first objective was to understand to what extent a coculture of DDCCs and AT1-like lung epithelial cells could recapitulate pathways and processes observed in vivo in the two populations. To do so, we derived the total RNA of D2.0R cells (a model of DDCCs) from different conditions: monoculture on plastic, coculture with AT1-like cells and DDCCs isolated from mouse lungs [9]. In parallel, we also isolated and purified total RNA from AT1-like cells in monoculture and after a coculture with DDCCs. We then performed RNA sequencing of the purified samples and a GSEA of different pairs of samples (outlined in Figure 1A). We first compared the RNA sequencing from AT1-like cells in the monoculture to that of the AT1-like cells cocultured with DDCCs. Our transcriptomic analysis of cocultured AT1-like cells revealed that the DDCCs profoundly affect several signaling and metabolic pathways in lung epithelial cells. We detected downregulation of several signaling (Wnt, TGFβ, PI3K, Notch) and metabolic pathways (glycolysis and lipid metabolism) as well as an activation of electron-transport chain process and proliferation gene sets (Figure 1B). The induction of a proliferative response in lung epithelial cells by disseminated cancer cells was of particular importance as it reinforced our previous findings in vitro and in vivo, where we observed proliferation in AT1 cells directly contacting DDCCs with multiple techniques [9].

We then turned our attention to the analysis performed in DDCCs and compared the RNA sequencing from D2.0R cells. Remarkably, there was a highly significant correlation between genes regulated in the coculture with those from lung-disseminated cells (Figure 1C, left). Moreover, the correlation became even stronger when the pathways and processes were compared rather than single genes (Figure 1C, right). For example, proliferative signatures were downregulated in cocultured DDCCs, in line with what we previously observed with proliferation markers in vitro and in vivo [9]. This indicates that our coculture faithfully modeled processes observed in vivo both in DDCCs and lung epithelial cells.

**Figure 2:**
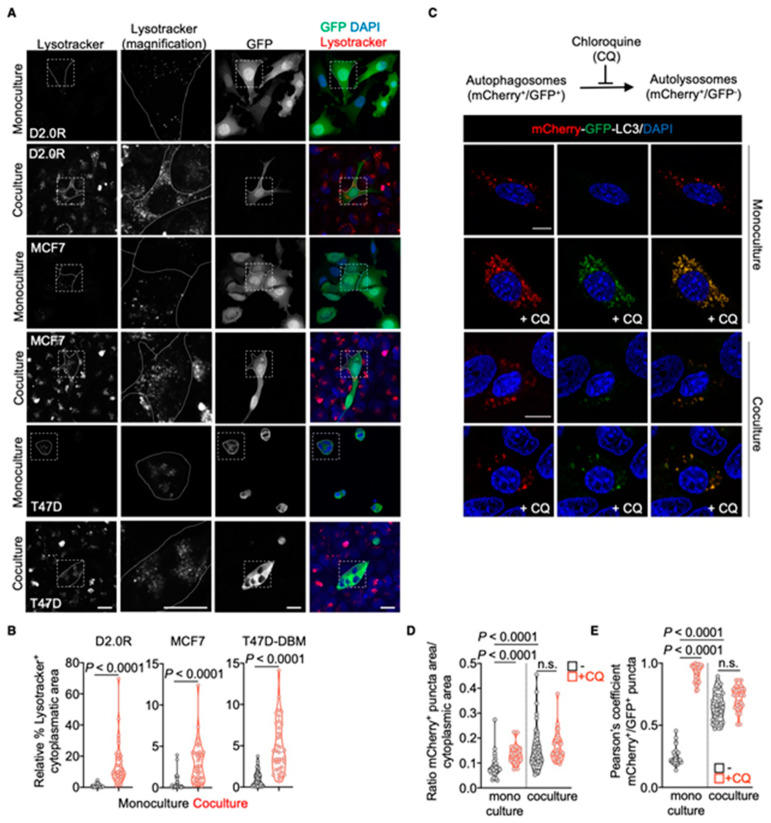
Accumulation of lysosomal vesicles in cocultured DDCCs. (**A**) Lysosomal accumulation in D2.0R-EGFP, MCF7-EGFP and T47D-DBM-EGFP cells upon monoculture or coculture with AT1-like cells as visualized by Lysotracker staining. Dashed box: area magnified in the middle images. Scale bar: 20 μm. (**B**) Quantification of the relative cytoplasmic area with Lysotracker+ staining. Mann-Whitney test. *n* = 3 independent experiments. (**C**) Representative pictures of mCherry and GFP fluorescent signals in D2.0R cells transfected with mCherry-GFP-LC3 tandem construct upon monoculture or coculture. Scale bar: 20 μm (monoculture), 15 μm (coculture). (**D**) Cytoplasmic area with mCherry+ve puncta in cells showed in (**C**). Kruskal–Wallis test, multiple comparisons. *n* = 2 independent experiments. (**E**) Colocalization of mCherry+ve and GFP+ve signals in cells showed in (**C**) expressed with Pearson’s correlation coefficient. Kruskal–Wallis test, multiple comparisons. *n* = 2 independent experiments.

Gene signatures related to lysosomal biogenesis and vesicle transport stood out as processes significantly upregulated in disseminated cancer cells in vivo and upon coculture with AT1-like cells (Figure 1D). We then looked at lysosomal vesicles in mouse and human models of DDCCs alone or in coculture with AT1-like cells. To do so, we took advantage of the Lysotracker probe, which consists of a fluorophore linked to a weak base. This compound is freely membrane permeant at natural pH and effectively labels acidic compartments, such as lysosomes, in live cells. In line with the hypothesis from transcriptome analysis, lysosome accumulation was observed in different human and mouse DDCCs in coculture compared to monoculture (Figure 2A,B). Of note, both monocultures and cocultures were maintained in a mitogen-low, nutrient-low medium (MLNL) and thus, a basal staining of the lysosomal compartment was visible. Nevertheless, the coculture with AT1-like cells significantly increased the accumulation of lysosomes (Figure 2A,B).

We next asked whether an increased lysosomal compartment was a consequence of increased autophagic-flux. To do so, we employed the tandem fluorescent reporter mCherry-GFP-LC3 [16]. LC3 is a vertebrate homologue of ATG8, one of the core genes of the autophagic machinery, and is associated with pre-autophagosomal structures, autophagosomes and autolysosomes [18]. During autophagosomes formation, fluorescence of GFP and mCherry was visible, and autophagosomes were visible as yellow spots. When autophagosomes fused with lysosomes, forming autolysosomes, the internal pH of the vesicles dropped, quenching GFP fluorescent signal, while leaving unaltered the red signal from mCherry [19]. Autolysosomes thus appeared as red dots, while autophagosomes were yellow dots. As shown in Figure 2C–E, DDCCs in monoculture show predominantly red vesicles compared to yellow vesicles, indicating low accumulation of autophagosomes. To our surprise, cocultured DDCCs showed an increased number of mCherry+ve spots highly overlapping with GFP+ve puncta (Figure 2D,E). An increased number of autophagosomes can be the result of opposite effects on autophagic-flux: induction of autophagosomes formation or block of autophagosomes maturation to autolysosomes. To distinguish between the two, we treated DDCCs with 100 μM chloroquine, which impairs lysosomal acidification and thus autophagosome degradation after phusion with lysosomes. Interestingly, while chloroquine increased the number of yellow puncta, i.e. autophagosomes, in monocultured DDCCs, it didn’t alter the autophagic flux in cocultured DDCCs (Figure 2C–E). This indicated that the autophagic flux was already blocked in cocultured DDCCs and macroautophagy cannot account for the observed lysosomal accumulation.

**Figure 3:**
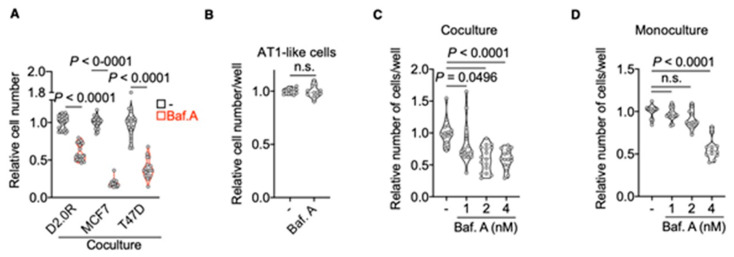
DDCCs are sensitive to inhibition of lysosomal function. (**A**) Relative cell number of the indicated indolent breast cancer cells upon coculture with AT1-like cells, after treatment with 4 nM Bafilomycin A1 for 4 days. *n* = 4 for D2.0R, *n* = 3 for MCF7 and T47D-DBM cells (independent experiments). Dunn’s test. (**B**) Mean normalized cell viability, assayed by resazurin staining, of AT1-like cells treated or not with 4 nM Bafilomycin A1 for 4 days. *n* = 3 independent experiments. Two-tailed *t*-test with Welch correction. (**C**) Relative number of D2.0R cells in coculture, after 4 days of treatment with different doses of Bafilomycin A1 or control treatment. *n* = 3 independent experiments. Kruskal–Wallis test. (**D**) Relative number of D2.0R viable cells in monoculture after 4 days of treatment with different doses of Bafilomycin A1 or control treatment. *n* = 3 independent experiments. Kruskal–Wallis test.

Having established an in vitro system that mimics lung-specific, in vivo processes upon dissemination of DDCCs, we asked whether functional lysosomes were required for the survival of DDCCs in this context. Blocking lysosome acidification with Bafilomycin A1 led to the reduced survival of human and mouse DDCCs in the coculture without affecting epithelial cells (Figure 3A,B). We then compared sensitivity to Bafilomycin A1 of DDCCs in MLNL medium in the monoculture or in coculture. Importantly, the coculture with AT1-like cells increased the DDCCs sensitivity to inhibition of lysosomal acidification (Figure 3C,D). As lysosomal flux is a core process of each cell, all the cells were expected to be sensitive to the inhibition of lysosomal function to some extent. That said, our results indicated that increased lysosomal accumulation in cocultured DDCCs was accompanied by increased sensitivity to the lysosomal acidification blockade compared to DDCCs cultivated alone.

**Figure 4:**
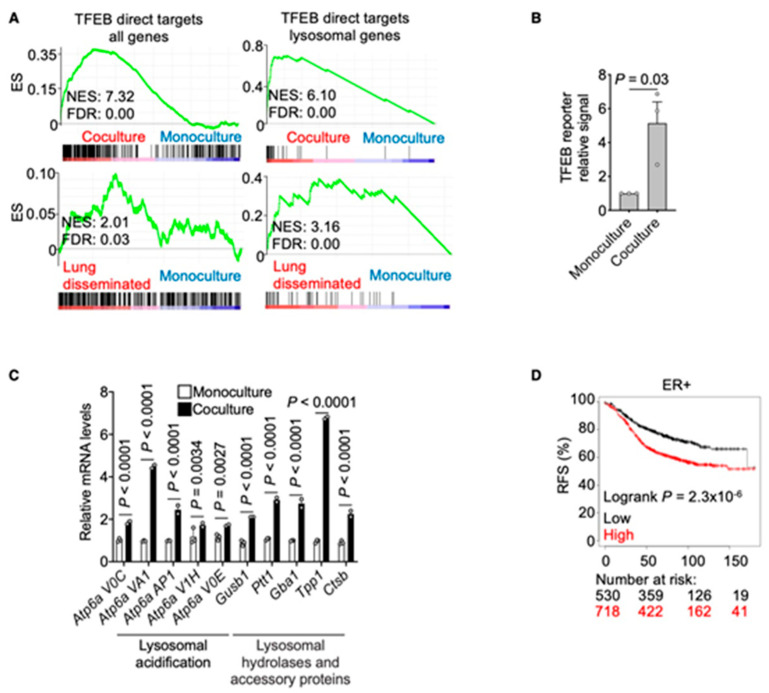
Transcription factor EB (TFEB) transcriptional response is activated in cocultured DDCCs (**A**) Profile of the running ES score for gene sets including TFEB direct targets (all targets or subselection of lysosomal genes, Table S1) after Gene Set Enrichment Analysis (GSEA) of D2.0R-EGFP cells disseminated in vivo, in coculture, or monoculture as indicated. (**B**) Relative induction of transfected TFEB-luciferase reporter in D2.0R-EGFP cells cocultured with AT1-like cells compared to monoculture. *n* = 3 independent experiments, ratio paired two-tailed *t*-test, mean with SEM. (**C**) Relative induction of TFEB direct lysosomal targets in D2.0R-EGFP cells cocultured with AT1-like cells compared to monoculture. *n* = 2–3 independent experiments. Two-way ANOVA, multiple comparisons. (**D**) Kaplan–Meier curve showing Relapse-Free Survival of ER+ breast cancer patients derived from the database at https://kmplot.com/analysis/ (accessed on 27 February 2021), stratified according to the TFEB signature (Table S2).

TFEB, a member of the MiT-TFE family of transcription factors, is a master regulator of lysosome biogenesis and associated metabolic processes [20,21,22]. To gain insight into its role in the activation of lysosomal compartment in our different conditions, we queried our transcriptomic analysis with a gene list of 471 TFEB direct targets and with a selection of TFEB direct targets with known lysosomal function [23,24]. Both signatures were highly enriched in DDCCs in vivo (lung-disseminated) and cocultured dormant cells, suggesting that activated TFEB might be responsible for the observed lysosomal accumulation (Figure 4A). To investigate this hypothesis experimentally, we took advantage of a luciferase reporter containing CLEAR sites, that is, the DNA consensus sites predominantly found in the promoter regions of autophagy-lysosomal genes and recognized by TFEB and the other members of MiT-TFE family [23,24]. D2.0R cells were first transfected with the luciferase reporter and then cultivated in monoculture or coculture before being analyzed for luciferase expression. As shown in Figure 4B, TFEB transcriptional activity was increased in DDCCs upon coculture with AT1-like cells compared to DDCCs alone. Moreover, several endogenous TFEB lysosomal target genes were enriched in coculture compared to monoculture (Figure 4C). So far, results have shown an increase in TFEB transcriptional response and lysosomal accumulation in DDCCs in vivo and in coculture with lung epithelial cells. We also showed that inhibition of lysosomal accumulation led to increased cell death of DDCCs in coculture. To test whether TFEB transcriptional response correlated with increased persistence of DDCCs in patients, we queried publicly available datasets of gene expressions from breast cancer patients with a signature of TFEB direct targets and observed that higher expression of the gene signature in ER-positive breast cancer patients was significantly linked with the increased likelihood of relapses (Figure 4D). These data suggest the activation of TFEB-dependent lysosomal biogenesis in DDCCs in vivo and in a lung coculture system and support its involvement in survival and persistence of disseminated indolent breast cancer cells in patients.

Delayed relapses of metastatic cancers are a significant hurdle in cancer therapy. While some types of cancers, such as lung and colon cancers, metastasize shortly after the detection of the primary tumors, other cancers (e.g., breast and prostate as well as melanoma) persist as indolent undetectable metastatic lesions for many years before reawakening [3,5]. In particular, ER-positive breast cancers showed increased propensity for late relapses compared to ER-negative subtypes [4]. The development of eradication therapies for DDCCs while still in a quiescent phase, will significantly benefit the survival of cancer patients. As disseminated indolent cells are characterized by limited growth, the accumulation of genetic lesions is unlikely to explain the shift in their behavior. Moreover, the role of the microenvironment, as well as of systemic signals, are likely to significantly influence the recurrence of dormant metastatic cancer cells. Recent evidence points towards dissemination strategies that proceed in parallel with the development of the primary tumor [25,26]. Due to the asymptomatic nature of DDCCs, the detection and isolation of those cells in patients is still technically and ethically unfeasible except for cells disseminated in the bone marrow [2,3]. For this reason, the development of in vitro systems that faithfully recapitulate features of the DDCCs’ microenvironment crosstalk is of paramount importance [5,6,27]. Here, we present evidence that a simple coculture of DDCCs and lung stromal cells is sufficient to induce a transcriptional response largely overlapping with the one obtained from in vivo isolated DDCCs (Figure 1C). Moreover, this overlap is not limited to DDCCs, as cocultured lung epithelial cells showed the activation of proliferative pathways, which agreed with the increased proliferation observed in AT1 cells in vivo [9]. Starting from this evidence, we interrogated our transcriptomic analysis and identified the TFEB-lysosomal pathway as the main transcriptional response activated in vivo and in coculture in DDCCs. This result was experimentally validated by staining cocultured DDCCs with a lysosome-specific dye and with a TFEB transcriptional luciferase reporter. Although our in vitro system does not encompass all the components of the in vivo metastatic niche, it includes the presence of organ-specific epithelial cells (as AT1 cells cover the majority of distant lung surface), cellular density and a low DDCC-epithelial cells ratio, low mitogenic culturing medium and ECM deposition (over several days of culture). Importantly, metastatic dormancy and reawakening is a paradigmatic example of the role of tumor heterogeneity. Indeed, the majority of disseminated aggressive breast cancer cells, don’t proliferate upon lung-dissemination, and they show markers for growth arrest [28]. The cellular model system here employed, D2.0R cells, is morphologically and phenotypically heterogeneous, so it could be exploited in the future to study how genetic and microenvironmental factors affect the emergence of aggressive clones.

Lysosomes are membrane-enclosed acidic organelles that serve as endpoints of several endocytic pathways as well as of self-catabolic processes such as apoptosis. Far from being mere digestive structures, lysosomes are a part of a network that senses nutrient availability and cellular requirements and coordinates responses to signaling pathways, metabolic adaptation, stemness, migration, proliferation and cell death [21,22]. Multiple pathways funnel into lysosomal compartments: caveolin-dependent and -independent endocytosis, phagocytosis and macropinocytosis. Hydrolases within the lysosomal compartment break down macromolecules such as lipids, proteins, carbohydrates and proteins into monomers that can be used as building blocks according to cellular need [22]. In this view, it’s not surprising that lysosomes have been shown to be essential for cancer cells in several contexts [22]. Aggressive cancer-cell proliferation relies on an adequate supply of extracellular nutrients, which is often impaired by a lack of mature blood vessels within the tumor [29]. Thus, scavenging for macromolecules from the microenvironment becomes an essential strategy for cancer cells to support their survival and growth. That said, uncontrollable cell growth is not a feature of DDCCs, at least during the prolonged quiescent period. Interestingly, it has been recently shown that enhanced lysosomal flux is essential for quiescence and stemness of neural and hematopoietic stem cells [30,31], via the control of epidermal growth factor receptor (EGFR) and glycolysis, respectively. This suggests that the requirement of lysosomal activation, beyond nutrient supply, is likely cell- and context-dependent. The role of lysosomes in DDCCs has never been directly addressed; however, two works have presented conflicting results on the role of autophagy [32,33]. Our results with lysosomal inhibitor Bafilomycin A1 support a positive role for autophagic-lysosomal flux in the survival of DDCCs (Figure 3A,C), while results in Figure 2C–E suggest that recycling and degradative routes other than macroautophagy must account for the observed lysosome accumulation. Importantly, our results showed that increased lysosomal accumulation makes DDCCs more sensitive to lysosomal inhibition compared to cells cultivated on plastic (Figure 3C,D). Thus, this simple coculture might be used as a platform for the screening of compounds aimed at eradicating lung-disseminated metastatic breast cancer cells.

## 4. Conclusions

Our data showed that a coculture of DDCCs with epithelial lung cells is sufficient to induce transcriptional processes significantly overlapping with those observed in DDCCs isolated from mouse lungs. From this analysis we identified the TFEB–lysosomal axis as the most significant pathways activated in DDCCs in vivo and in coculture, suggesting a role in the survival of disseminated cancer cells. As support for this hypothesis, cocultured DDCCs showed increased sensitivity to a lysosomal blockade compared to cells in monoculture and increased activation of a TFEB transcriptional reporter.

## Data Availability

RNAseq data have been deposited at the Gene Expression Omnibus with accession number GSE162440.

## Supplementary Materials

The following are available online at https://www.mdpi.com/2072-6694/13/5/1007/s1, Table S1: Gene sets added for GSEA, Table S2: TFEB direct targets list, Table S3: qPCR oligos used in the study.
